# Human and computer estimations of Predictability of words in written language

**DOI:** 10.1038/s41598-020-61353-z

**Published:** 2020-03-10

**Authors:** Bruno Bianchi, Gastón Bengolea Monzón, Luciana Ferrer, Diego Fernández Slezak, Diego E. Shalom, Juan E. Kamienkowski

**Affiliations:** 1 Laboratorio de Inteligencia Artificial Aplicada, Instituto de Ciencias de la Computación, Facultad de Ciencias Exactas y Naturales, Universidad de Buenos Aires - Consejo Nacional de Investigación en Ciencia y Técnica, Ciudad Autónoma de Buenos Aires, Argentina; 2 Departamento de Computación, Facultad de Ciencias Exactas y Naturales, Universidad de Buenos Aires, Ciudad Autónoma de Buenos Aires, Argentina; 3 Departamento de Física, Facultad de Ciencias Exactas y Naturales, Universidad de Buenos Aires, Ciudad Autónoma de Buenos Aires, Argentina

**Keywords:** Computational models, Language

## Abstract

When we read printed text, we are continuously predicting upcoming words to integrate information and guide future eye movements. Thus, the *Predictability* of a given word has become one of the most important variables when explaining human behaviour and information processing during reading. In parallel, the Natural Language Processing (NLP) field evolved by developing a wide variety of applications. Here, we show that using different word embeddings techniques (like Latent Semantic Analysis, Word2Vec, and FastText) and N-gram-based language models we were able to estimate how humans predict words (cloze-task Predictability) and how to better understand eye movements in long Spanish texts. Both types of models partially captured aspects of predictability. On the one hand, our N-gram model performed well when added as a replacement for the cloze-task Predictability of the fixated word. On the other hand, word embeddings were useful to mimic Predictability of the following word. Our study joins efforts from neurolinguistic and NLP fields to understand human information processing during reading to potentially improve NLP algorithms.

## Introduction

In everyday tasks our brain performs predictions about future events, anticipating possible future actions, and building an internal model of the environment that is not sensed directly. This active sampling of information is a continued and involuntary process^1–3^. Understanding brain processes that underlie predictions under a certain environment could shed light over the general processes of prediction in every environment. In particular, in reading tasks these predictions are operationalized in a variable called *Predictability*, which is defined as the probability of knowing a word before reading it. Predictability has been used widely in the neurolinguistic field to understand the variation of gaze duration (GD) over words in eye tracking experiments^4–7^.

In sentence-reading studies, Predictability correlated inversely with the processing time of the word, which was measured using eye movement variables like first fixation and gaze duration, or skip probability,^4,7^ and self-paced reading times^8^ (see Holmqvist *et al*.^9^ for more details on eye movements and eye tracking measures). This was interpreted as an increase in processing cost for low-predictability words. In addition, there is some evidence, also from eye movements in sentence reading tasks, that the Predictability from the upcoming word (N + 1) can influence the gaze duration on the foveated word (i.e. the word that is being read). This effect, which is usually referred to as "*parafoveal-on-foveal word effect*” or more generally “*successor word effect*”, raises the debate as to whether this is evidence of parallel processing over more than one word, as it is stated by Kliegl *et al*. in a study analysing nine previous experiments^10^, or just a confounding factor over the serial hypothesis, as discussed by Rayner *et al*.^11^. It is worth stressing that these are all sentence-reading studies, but the implications of Predictability could be really different in longer text reading, where different sources of Predictability could be drawn from the larger previous context.

In these studies, Predictability is estimated with an independent experiment, from responses on a separate group of humans than those that performed the reading task. Thus, despite its simple definition, estimating Predictability is usually an expensive procedure. The experiment used for this is called cloze-task^12^. It consists of a survey in which subjects read incomplete texts and guess missing words according to their beliefs on the most probable words given the preceding context. The proportion of correct answers for each word is defined as its Predictability or *cloze-Predictability* from now on. To estimate cloze-Predictability accurately, it is necessary to use a large number of participants that have to read and complete the missing words for exactly the same material that is going to be used in the eye movement experiment. This makes it very difficult and expensive to change the corpus from one experiment to another, which is recommended to obtain better generalization and reproducibility of the results. Having a computational estimation of cloze-Predictability would be a great step forward for neurolinguistics, not only from the methodological point of view, but also to enable researchers to experiment with different contributions of the components of the computational model. This would allow us to better understand the sources of the effect of this variable that is involved in the prediction of an active sampling of the visual world^1–3^.

From the Computer Science field, and Natural Language Processing (NLP) field in particular, there is also a large interest in predicting upcoming words from the context. In recent decades many approaches have been applied to develop algorithms that are able to predict upcoming words in a given text. Nevertheless, these predictions are usually evaluated against the original word in a test corpus, without taking into account how humans predict words and how these predictions affect cognitive processing itself; for instance, eye movements or pupil dilation are often used as proxies. In the present work, we used different versions of four widely used algorithms (*N-grams*, *Latent Semantic Analysis* (LSA), *Word2Vec* and *FastText*) to build different computer-based Predictability models (*computer-Predictability*), and we used them as replacement for cloze-Predictability in a statistical analysis of gaze duration.

Briefly, the N-gram algorithm is based on the estimation of the probability of finding an exact chain of N contiguous words in a training corpus. For example, the computer-Predictability for ‘*sugar*’ in the sentences ‘*I like coffee with sugar*’ could be estimated roughly using N-gram with *N* = 5 (i.e., 5-gram) as the ratio of the number of appearances of this exact chain of five words in the corpus over the number of appearances of ‘I like coffee with*’, where * is any word. Different variants of this simple idea have appeared through the years that deal with different normalization and smoothing procedures (i.e., how to deal with missing sequences, which is very common for large N). Note that if N is too big, not only the first term will become zero, but also the second one, which means that the (N − 1) previous words did not appear together in the training corpus. To solve this issue, when the exact context is not present in the training corpus, the (N − 1)-gram could be used with a proper normalization for the change in N (‘*drink coffee with sugar*’ in this example). Furthermore, very interesting extensions include, for instance, the *Factorial N-grams* and the *cache N-grams*. The *Factorial N-grams* provide an algorithm for considering not only the sequence of given words but also their roots, number, gender, or their semantic categories. The *cache* extension of the N-gram model takes into account the recently incorporated information, by training a separate N-gram model using the text read up to the present word^13,14^. It is common to use just a 1-gram for this separate model because there is very few material to estimate higher N-grams. Then, it is combined with the N-gram estimated in a large independent corpus, typically by linearly interpolation (see *Method*^13,14^ *s* > *Computer predictions* > *N-grams*).

Another very important class of algorithms developed in the NLP field are those that have *word embeddings* as an outcome. The embedding of a word is its representation in a multidimensional space. This idea was introduced in the 1950s, when some linguists stated that the meaning of a word is its use in the language, and that generating multidimensional space where similar words were neighbours could be useful to understand semantic relations between words^15^. Using mathematical representation for words allowed for operations to be performed with the resultant vectors, like estimating "semantic closeness” between two words by calculating the cosine distance between their vectors. Here, we propose to use the mean distance (cosine similarity, *CS* from now) between the target word and each of the context words (in a given vectorial representation) as the computer-Predictability. We hypothesize that this computer-Predictability is a partial estimation (using only semantic information) of Predictability.

In the present work we tested three different word embeddings. On one hand, we calculated a word-by-document co-occurrence matrix in LSA algorithm^16^ where each cell contained the frequency of each word *w*_*i*_ in each document. Because this resulted in a very sparse matrix (i.e., many words did not appear in many of the documents), its dimensionality was then reduced to *d* dimensions using Singular Value Decomposition. The resulting words-by-d matrix contained a vectorial representation for each word, and the d-dimensional space represented the semantic space.

On the other hand, Word2Vec^17^ represents a more modern approach by using neural networks to generate the embedding. This algorithm consists of a fully connected neural network with one hidden layer with *d* nodes. This network is trained to predict words given the preceding and future context in which they appear in a corpus. The hidden layer activation for each word in the corpus is then used as its own embedding. As in LSA, this results in a d-dimensional semantic space where words are represented by vectors. The third algorithm, FastText^18^, relies on the same architecture, but it implements an additional training, where character-bigram is used as a feature. This aims to capture sub-lexical information and allows it to represent words outside the training corpus.

By pulling together these successful computational methods with the eye movement data and models from cognitive science, we aimed to understand the different computational calculations executed by the brain when trying to predict the upcoming word. In particular, we wanted to know how this prediction and the prediction of the next word affect present fixation. Moreover, this is a step forward in using better proxies for human cognitive processes during reading, such as eye movements or pupil dilation, to evaluate NLP algorithms. Here, we explore the parameters of these algorithms, such as N value and the use of cache in the N-grams, the number of words considered as context in the different embeddings, and how to represent the context mathematically. We also tested their impact as explanatory co-variables of eye movements compared with the cloze-Predictability estimated with human responses.

## Results

**Figure 1:**
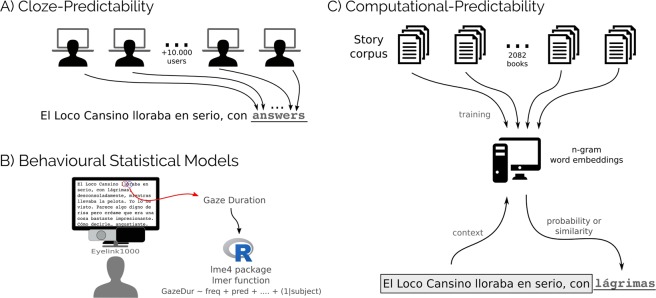
Experimental designs: (**A**) The human Predictability was estimated from the online responses of several participants to a web cloze-task experiment. Each participant had to complete one of every 30 words, and the text was uncovered as they responded. (**B**) Eye movements were recorded in separate participants that read three of the eight texts in the lab. The eye movement measures (Gaze duration) were analyzed using Linear Mixed Models. (**C**) Computational algorithms were trained on a large corpus of texts from a similar domain as the tested short stories (**A**,**B**). Image sources (**B**) R project (https://www.r-project.org/logo/, The R Foundation, CC BY-SA 4.0 https://creativecommons.org/licenses/by-sa/4.0/, no changes made).

**Figure 2:**
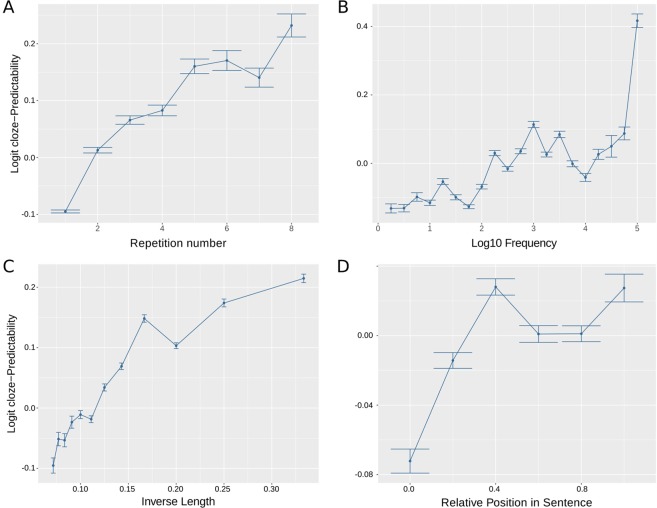
Analysis of the cloze-Predictability. It co-varied with several properties of the word (**A**) the repetition number within the text, (**B**) the (*log*_10_) frequency in the lexicon, (**C**) the (inverse) length, and (**D**) the relative position within the sentence. Lines represents the mean (*logit*) cloze-Predictability, and the error bars are the standard error of the mean.

Predictability was first estimated by humans’ responses to a cloze-task. Approximately 2500 participants read 1–8 texts (mean = 1.92) and completed approximately 300 words out of 26366 unique words, where each participant completed one every 30 words in an online platform (Fig. 1A). Correlations between (logit) cloze-Predictability with the repetition number, the (log) frequency in a corpus, and the (inverse of) word length (Fig. 2) showed the expected behaviour (i.e., the more frequent, the shorter, and the more repeated the words were inside the text, the more predictable the words were)^19^.

A separate set of 36 participants performed an eye-tracking experiment in the lab. Each participant read three of the eight texts. Texts were assigned to participants pseudo-randomly (Fig. 1B). Finally, we trained different computational models drawn from the Natural Language Processing (NLP) field in a larger corpus. This corpus was also composed of short stories in Spanish. The original stories were not contained in the larger corpus (Fig. 1C).

**Figure 3:**
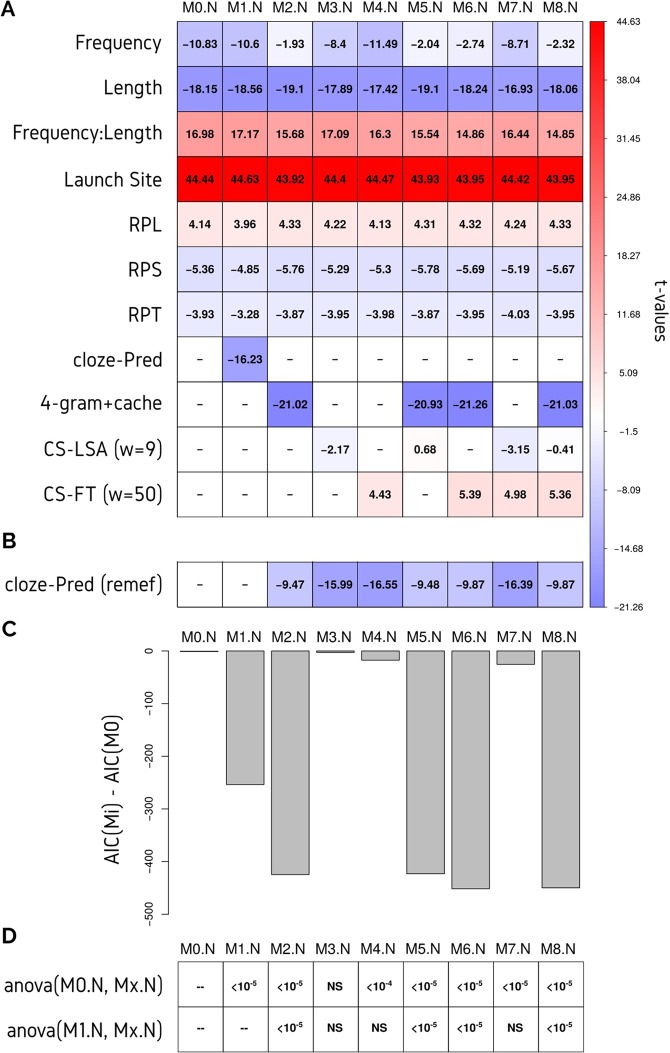
LMM analysis of the Gaze duration. (**A**) Different models includes the cloze-Predictability of the word N and different combinations of the NLP algorithms: 4-gram+cache, LSA (with a context of nine words) and FastText (with a context of 50 words). See Supplementary Table S1 for estimates and confidence intervals. (**B**) A new LMM with the cloze-Predictability was applied to the Gaze Duration with the fixed effects of each given model (columns) removed. (**C**) The difference between the AIC for each model was compared with the AIC of the baseline model (M0). Blueish colours represent negative t-values and reddish colours represent positive t-values. The exact t-values are also included in each cell. (**D**) p-values from ANOVA comparison between each model with the baseline model (M0.N) and with the cloze-Pred model (M1.N).

The computational and cloze-Predictability effects on gaze duration (First Pass Reading Time) were analyzed using Linear Mixed Models (LMM) (Fig. 3, Supplementary Table S1). First, a baseline model (**M0.N**) was generated. This model included six fixed effects and one interaction that were all from the fixated word (N): saccade launch site (*Launch Site*), word length (*length*), word frequency (*freq*), relative position in line (*rpl*), relative position in text (*rpt*), relative position in sentence (*rps*), and the interaction between *length* and *freq* (*length:freq*). See methods for details on how these variables were calculated. All these fixed effects were analyzed previously in Kamienkowski *et al*.^20^. The addition of these fixed effects from the previous and next words^7^ did not cause any major change to the fitted models. Next-word cloze-Predictability (*cloze-Pred N + 1*) was used as a fixed effect to better understand computer-Predictability. The models described below, were compared with this baseline model (**M0.N**) using the Akaike Information Criterion (AIC) and ANOVA tests as implemented for the *merMod-class* in the *lme4* package. The *anova()* function tests the log-likelihood ratio between the specified models^21,22^. The AIC takes into account the number of effects but it only evaluates the relative performance between models rather than the absolute performance of the model. In general, the more negative the AIC is, the better the model explains the dependent variable (in this case, gaze duration).

The cloze-Predictability was included in **M1.N**. As expected, this variable showed a strongly significant effect on gaze duration (Fig. 3A, Supplementary Table S1). Moreover, the model showed a large decrease in the AIC in relation to the baseline model (Fig. 3C) that resulted highly significant (Fig. 3D).

Computer estimations for predictability were evaluated one at a time by replacing the cloze-Predictability (**M2.N** to **M4.N**). We first explored the parameter space for the N-gram+cache predictabilities, and we decided to use *N* = 4, *δ* = 0.00015 and *λ* = 0.15 (see Supplementary Fig. S1). The resulting co-variable was included in the model (**M2.N**, *N-gram+cache model*), which showed a very significant contribution (Fig. 3A, Supplementary Table S1). Interestingly, the introduction of the *N-gram+cache* variable in the model generated a decrease in the significance of the *frequency* effect (turning it not significant), which suggested a correlation between these two variables. This occurred even though, the AIC for this model was significantly larger than the AIC for **M1.N**, which indicated a better fit (Fig. 3C,D).

Then, we explored the parameter space (i.e., the number of words considered for the context) for the different estimations that were based on embeddings. The metric based on the Cosine Similarity for LSA (CS-LSA) presented better results for a shorter window (*window* = 9) than the one for FastText (CS-FT, *window* = 50) (Supplementary Fig. S2). Given that the metrics for Word2Vec did not exhibit an stable behaviour within the windows range (i.e., t-values observed for small windows became significant or not significant with small changes in the analyzed corpus), it was excluded from further analysis (Supplementary Fig. S2C).

In contrast to N-gram+cache scores, for the CS-LSA co-variable (**M3.N**, *window* = 9) the effect was only slightly significant, and it also generated a small drop in the significance of the frequency effect, which did not prevent it from being significant (t-value goes from −10.83 to −8.4 and a similar behaviour is observed in the confidence intervals). Ong and Kliegl (2008) showed a result in the same direction when exploring the possibility of using conditional co-occurrence probability (CCP) as computer-Predictability. They concluded that methods similar to CCP were prone to have a bias towards high frequency words because they had a more reduced dynamic range than low frequency words (i.e., because they appeared in every document)^23^. But, more striking, the AIC showed a very small improvement relative to the baseline and dropped in performance relative to the **M1.N** (Fig. 3C) and the ANOVA test shows that this improvement is non significant (Fig. 3D). The CS-FT co-variable (**M4.N**, *window* = 50) showed a more significant effect, but it was surprisingly positive. Interestingly, the addition of this regressor did not affect the frequency effect. The AIC for this model showed a better fit than the model with CS-LSA, but still not as much as **M1.N** and **M2.N** (Fig. 3C). This is also seen in the ANOVA test that shows a significant difference with M0.N but non significant with M1.N (Fig. 3D).

To analyze how these three computer-Predictability estimations interacted within the LMMs, all the possible combinations were tested (**M5.N** to **M8.N**, Fig. 3A). The fact that CS-LSA effect vanished when N-gram+cache scores are present suggested that these two effects are overlapped (**M5.N** and **M8.N**, Fig. 3A, Supplementary Table S1). Conversely, CS-FT did not show overlapping (**M6.N** and **M8.N**, Fig. 3A, Supplementary Table S1). Finally, CS-LSA and CS-FT effects did not show major changes when analyzed together (**M7.N**, Fig. 3A, Supplementary Table S1).

To better understand how computer-Predictability algorithms mimic cloze-Predictability, residuals of each model from **M2.N** to **M8.N**, which were obtained by removing estimates of all the fixed effects, were used to fit a new LMM with the same random effect structure and cloze-Predictability as the only fixed effect. This was supported by the *remef()* function (see Methods for details)^20,24^. The difference between the significance of the cloze-Predictability effect in **M1.N** and its significance in the new model served as an indicator of how much our algorithms modelled it. Only models with the N-gram+cache score variable showed a large decrease in the cloze-Predictability t-value, which indicated that only this algorithm was capable of partially capturing the cloze-Predictability (Fig. 3B).

**Figure 4:**
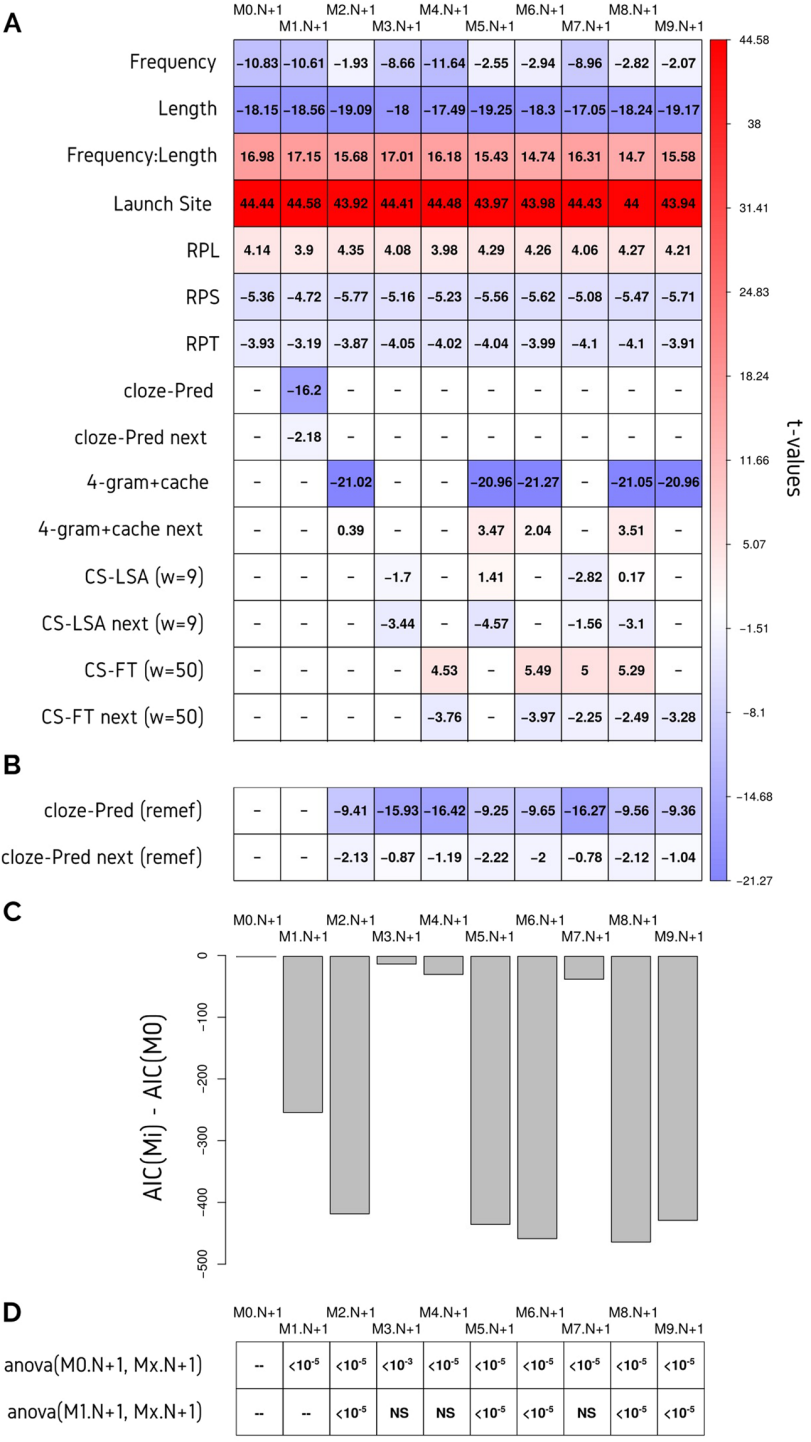
LMM analysis of the Gaze duration. (**A**) Different models includes the cloze-Predictability of the word N and N + 1, and different combinations of the NLP algorithms for the N and N + 1 (next): 4-gram+cache, LSA (with a context of nine words) and FastText (with a context of 50 words). See Supplementary Table S2 for estimates and confidence intervals. (**B**) A new LMM with the cloze-Predictability was applied to the Gaze Duration with the fixed effects of each given model (columns) removed. (**C**) The difference between the AIC for each model was compared with the AIC of the baseline model (M0). Blueish colours represent negative t-values and reddish colours represent positive t-values. The exact t-values are also included in each cell. (**D**) p-values from ANOVA comparison between each model with the baseline model (M0.N + 1) and with the cloze-Pred model (M1.N + 1).

To further investigate the capabilities of the tested algorithms, we tested the effects of computer and cloze-Predictability of the next word (N + 1) on gaze duration (Fig. 4, Supplementary Table S2). All models from the previous section were fitted again, using both, the Predictability of the fixated word (N) and the Predictability of the next word (N + 1). Cloze-Predictability of the following word showed a significant effect on gaze duration. Interestingly, in contrast to some of the previous studies^10,25^, this effect was negative. A similar result was seen when Chinese reading^26^ was analyzed but, to our knowledge, there was no evidence of a similar result in an alphabetic language. In our context, for short stories in Spanish, we obtained a negative effect of cloze-Predictability for the word N + 1.

In contrast to the results on the fixated word, the effect of the N-gram+cache score on the following word was not significant (**M2.N + 1**, Fig. 4A, Supplementary Table S2). Inversely, both embedding-based scores showed better performances for word N + 1: CS-LSA effect for word N + 1 was more significant in this model than in the model for word N **M3.N** (Fig. 4A, Supplementary Table S2). The effect of CS-FT on N + 1 was in the same direction than the cloze-Predictability of N + 1 (**M4.N + 1**, Fig. 4A, Supplementary Table S2). This was supported by the *remef()* analysis, where cloze-Predictability for N + 1 became not significant for **M3.N + 1** and **M4.N + 1** (Fig. 4B). Additionally, when computer-Predictabilities for the N + 1 word were analyzed together (**M5.N + 1** to **M8.N + 1**), they behaved completely differently from the effects for the fixated word. The N-gram+cache score became positively significant in the presence of CS-LSA and/or CS-FT, and CS-LSA and CS-FT seemed to account for the same variance. Altogether, these results suggested that different cognitive processes were related to the effect of Predictability on the fixated word and the upcoming word.

Additionally, a model that summed all these results was implemented, which used the N-gram+cache for the fixated word and CS-FT for N + 1 (**M9.N + 1**, Fig. 4A, Supplementary Table S2). This model resulted in an AIC close to the best of all the explored models with only two co-variables included over the baseline model (Fig. 4C) and significantly better than M0.N + 1 and M1.N + 1 (Fig. 4D). It produced a huge decrease in significance of the cloze-Predictability effect for the fixated word (Fig. 4B). Also, it caused the effect of the cloze-Predictability of word N + 1 on gaze duration to become not significant (Fig. 4B).

## Discussion

In the present study, we aimed to investigate computational estimations of word predictability and to analyze how they can be used to model gaze duration (GD) during reading of long texts. To our knowledge, there is little evidence on the Predictability effect for long texts (~3000 words)^27^ and, even more, there are no previous studies in Spanish. This could be due to the difficulty and the resources needed to perform a cloze-task (i.e., the experiment commonly use to estimate word Predictability) for all the words in this type of corpus. We worked on a corpus of eight stories with more than 25,000 words, and we implemented a cloze-task with thousands of participants. These data are now available for further research. The estimated cloze-Predictability from this corpus followed the expected behaviour in terms of correlations with other relevant variables, such as the lexical frequency, the length of the words, and the repetition number within the text.

It is worth mentioning that in the literature, cloze-task is not conducted consistently in all neurolinguistic studies, and it depends mainly on the material. For instance, when sentences or short paragraphs are analyzed, participants are asked to predict every word, one by one throughout the text. Even more, when only a target word is relevant, they are asked only for that word. In the present study, to make the experiment feasible, cloze-task participants answered one out of every 30 words throughout the entire text (around 100 words per participant per text). Thus, for each answer, subjects counted with plenty of information about the target word; subjects had a great deal of semantic information about the text, the writer’s style, and so on. Furthermore, they were potentially not biased by their previous responses. These differences implied that the prediction mechanisms captured by this cloze-Predictability were slightly different from those captured by the cloze-Predictability in isolated sentences. Also, in the reading task, the processes that underlie eye guidance through the text may be different between sentences and long texts. For instance, the low level processes that occurred at the word level could be shadowed by the high level processes that developed at the level of the integration of new information with information from the beginning of the text. We hypothesized that these differences, both in the estimation of Predictability and the in eye movements, were the reason for the negative relation between cloze-Predictability of the following word (N + 1) and GD on word N (Fig. 4, M1.N + 1). This negative relation was found previously only in Chinese sentence-reading^26^, but not in German or Spanish sentence-reading^10,25^.

Many Natural Language Processing (NLP) algorithms were designed to deal with the task of completing sentences, that focused on human writing, which is a slow and thoughtful process. Nevertheless, many human-computer interactions or computer-based human interactions are based on faster, more reactive interplay. To understand and mimic those cognitive processes, NLP algorithms must model more covert or implicit measurements of human thought. Eye movements may be a reliable window into the human mind^28^ because they are usually involuntary and reflect cognitive demands. It would be interesting to train NLP models using all of this information together. One example would be to tune Word2Vec-like embeddings to incorporate information that allows us to predict gaze duration. This would potentially change the focus from the writer to the reader’s expectations. In the present study, we performed a step forward into the integration of NLP algorithms and reading studies using eye movements. Specifically, we analyzed computer estimations of word Predictability with four different algorithms: *N-gram*, *LSA*, *Word2Vec*, and *FastText*. A 4-gram model was used with the addition of the local word frequency (see Supplementary Fig. S1). LSA, Word2Vec, and FastText were studied using 300 dimensions and average Cosine Similarity (CS) with the previous words (context) as a proxy for Predictability, which used different context sizes (see Supplementary Fig. S2).

The estimation of the impact of these algorithms was analyzed using Linear Mixed Models (LMMs) and the Gaze Duration as the dependant variable (Fig. 3, Supplementary Table S1). The results of each of these computer-based Predictabilities on the gaze models clearly showed that the one that best explained eye movements was the N-gram+cache, even though it generated a large decrease in the frequency effect, presumably because of the high correlation between these two variables. In comparison with word-embeddings, the N-gram+cache model has the advantage of capturing the complexity of writing and not only addresses for semantic information. Nevertheless, it was strongly limited because it used the probability of an exact chain of words that appeared in a corpus.

Computational models also explained the effect of the Predictability of word N + 1 on GD (Fig. 4, Supplementary Table S2). On the one hand, the N-gram model correlated with the GD in the same direction that the cloze-Predictability used in traditional psicolinguistics studies in sentences (i.e., negative effect of the word N and positive effect of the word N + 1 in computer-based Predictibilities). This model, even with the addition of the local frequency information (*cache*), revealed more about the preceding short-term context of the word. On the other hand, word embeddings were more likely to capture long-term effects. Accordingly, they behaved more similar to our estimation of cloze-Predictability in long texts (i.e., negative for word N and for word N + 1, when included in the LMMs). This difference between N-gram+cache model and the word embeddings is consistent with the hypothesis that the effect of human predictability on eye movements is driven by different sources. In our case, the predictability on word N + 1 in long texts seems to be related to the semantic context, while in isolated sentences it is more supported by the immediate word context (both semantic and grammatical).

In summary, we used Eye Movements to understand not only the influence of classical linguistics variables but also the results from NLP algorithms. Eye movements, and also pupil dilation, served as measures of implicit or covert processing of the text, but responses or writing itself were measures of explicit or overt behaviours. This approach could serve to investigate the information that NLP algorithms capture from the text. It could lead to how this information influences human reading, and it could also be used to optimize some parameters, like the semantic space dimension, or even the training corpus, that could lead to better predictions. Here, we combined well-established and succesful NLP algorithms, like N-grams or LSA, with some newer approaches, like Word2Vec or FastText. But, the NLP field is changing very rapidly, and it would also be interesting to extend this approach to more modern algorithms, like the recently introduced Transformers^29^ (BERT^30^, ElMo^31^, ULM-FiT^32^, etc), that are based on Recurrent Neural Networks. The main issue with these algorithms is the high processing cost of the training. Nevertheless, it would be interesting to apply some ideas from these algorithms and to add implicit human responses (i.e. eye movements) for fine-tuning, which could optimize predictions with this information. Hence, this study is starting to open another bridge between Cognitive and Computer Sciences that has proven to be very successful for both sides in many other domains.

## Methods

### Eye Movement Recordings and Pre-processing

Data from eye movements during natural text reading was obtained from the **Buenos Aires Corpus**^20^. Thirty-six healthy subjects (11 women; age range 20–40 years; M = 24.9, SD = 3.8) participated in a 2-hour reading experiment. All participants were native Spanish speakers and had normal or corrected-to-normal vision. All participants were compensated with 4 American dollars for 2h of participation. All the experiments described in this paper were reviewed and approved by the ethics comittee: *Comité de Ética del Centro de Educación Médica e Investigaciones Clínicas* "*Norberto Quirno*” (CEMIC) and qualified by the Department of Health and Human Services (HHS, USA): IRb00001745 - IORG 0001315 (Protocol 435). All participants provided written informed consent in agreement with the Helsinki declaration. From the 10 original stories, eight were selected based on their length.

Gaze duration on every word was analyzed as a dependent variable in Linear Mixed Models (LMMs, see below). Short words (less than three letters) and the first and last word in sentences and lines were filtered out.

### Human predictions

#### Online Cloze-Task

Human predictability for every word of the eight stories was estimated using an online cloze-task (26366 total words). Participants logged into a web page and gave informed consent in agreement with the Helsinki declaration, declaring being older than 18 years old. The eight texts were assigned in pseudo-random order at the moment of the first login. Each presentation was divided into chunks of approximately 30 words. After every chunk, subjects answered the most probable following word. Cloze-Predictability was then estimated as the proportion of correct answers. Words with less than eight answers were not analyzed (19144 words left). On average, each word was answered 13 times (range: 8–37).

### Computer predictions

Four different models (LSA^16^, Word2Vect^17^, FastText^18^, and N-grams^33^) were trained using a corpus of 2082 Spanish books that consisted of >100 million words^34^. None of them included the stories used in the eye-movement and cloze-task experiments. In addition, FastText was tested using the pre-trained version with the Spanish Wikipedia^18^.

In the following subsections, computer-based Predictability models were presented, and their parameters were selected to analyzed their performance on all our eye movement corpora. Correlation and LMMs (with all the co-variables from the final analysis) were fitted. Conclusions drawn from these analysis only focus on the capacity of these NLP models to capture variance from Gaze Duration similar to cloze-Predictability. Further research should be done to generate predictive models (as they are known in Machine Learning) by building a larger corpus with cloze-Predictability and Gaze Duration data.

#### N-grams

The N-gram probability for each word in the stories from the Buenos Aires Corpus was calculated using the SRILM package (http://www.speech.sri.com/projects/srilm/). The window used to determine the context (N) was optimized using the correlation with the cloze-Predictability (Supplementary Fig. S1A). The optimal value for N was 4, after which the curve showed a plateau, which indicated that long chains of words did not appear in our training corpus.

To avoid getting probabilities equal to zero when a high-order N-gram is not available we used Katz smoothing (as it is implemented in the SRILM package). This backoff method uses the Good-Turing estimations^35^ to combine high- and low-order N-gram models. Briefly, the idea is to estimate the missing probabilities with lower N models, using the discount proposed by the Good-Turing estimation.

To compensate for the locality of this measure, as it only uses the last few words as context, and to generate a prediction that had some information about the text in which the word was embedded, we combine the N-gram estimated in a large corpus with a 1-gram estimated only in the text previously read (*cache model*). For this cache model, we used an Additive Smoothing^14^. Briefly, for every word *w*, it adds a small constant *δ* to the word count *c*(*w*), to prevent it from being 0. That is,

1 $${p}_{additive}=\frac{c(w)+\delta }{\delta V+N},$$

where *N* is the number of words and *V* is the vocabulary (i.e. the number of unique words).

We combine the N-gram and the cache models using a linear interpolation with a parameter *λ*,

2 $${p}_{ngram+cache}=\lambda {p}_{cache}+(1-\lambda ){p}_{ngram}.$$

Both *δ* and *λ* parameters were optimized for the 4-gram probabilities. We performed a grid search between *δ* = [0, 000050, 0005] and *λ* = [0, 050, 6], measuring the t-value of the 4-gram+cache variable in the **M2.N** model (Supplementary Fig. 1B). We kept the values of *δ* and *λ* with the maximum absolute t-value (*δ* = 0, 00015 and *λ* = 0, 15). It is important to note that the effect was largely significant throughout the whole grid, and that the t-values presented only small variations (between [−20 − 21]). Thus, although we learned these parameters in the same data used for testing, the main effect is present for a wide range of values for those parameters.

#### Latent Semantic Analysis (LSA) and Word2Vec

LSA^16^ and Word2Vec^17^ were trained using the python library Gensim to obtain 300 dimension vectors for each word of the corpus of stories. These vectors were used to determine the similarity (Cosine Similarity) between the target word and its previous context. The previous context was defined as the "with stopwords context”, where the *w* previous words were used, and the "without stopwords context”, where the *w* previous content words were analyzed.

To account for extremes in the frequency distribution *Term Frequency - Inverse Document Frequency* (tf-idf) statistics were performed before training LSA on 300 dimensions^15^. In Word2Vec, training words that appeared less than five times in the corpus were skipped, and the skipgram model was trained using 15 words on each side of the target word. This was only for Word2Vec training, not for the computational predictability estimation. All the other hyperparameters were used as defaults.

#### FastText

FastText is an implementation of the same algorithm that underlies Word2Vec (skipgram), which uses word and character ngrams to train the neural network. By doing this, it is supposed to account for more than just lexical information. A pre-trained data set with all the articles from Wikipedia in many languages is available online^18^. The Spanish dataset was used in the same way that it was used in Word2Vec.

#### Context window for word embeddings

Computer-Predictabilities for Word Embeddings were estimated as the cosine distance from the word *N* to the *w* previous words, by varying *w* between 1 and 150 (Supplementary Fig. S2). For LSA, two approaches were used. We calculated the distance to the resultant vector of the context (‘*LSA (Resultant)*’), and we calculated the mean distance between word N and each context word (‘*LSA (mean)*’). For Word2Vec and FastText, the built-in function ‘*similarity()*’ was used. LMMs were fitted with all the computer-Predictabilities generated, and the best computer-Predictability model was selected based on the correlation with cloze-Predictability, AIC, and t-value in the corresponding LMM.

### Linear Mixed Models (LMMs)

The *lmer* function included in the *lme4* package (version 1.1-8)^22^ was used to estimate fixed and random coefficients. Akaike information criterion (AIC)^36^ was used for model comparison with different fixed effects and identical random effects. To ensure a correct inter-model comparison, all of them were fitted using the exact same dataset. Because the *lmer* function removed the instances that had at least one missing value for any fixed effect only *complete cases* were analyzed. The AIC values (*AIC* = −2*logLik* + 2*nparam*, where *logLik* is the model likelihood and *nparam* is the number of parameters of the model) corrected the log-likelihood statistic for the number of estimated parameters and the number of observations to avoid overfitting during the process of model selection. Note that the AIC decreases with goodness of fit.

LMMs included a number of other covariates known to affect fixation duration. Launch site, position in text, sentence and line (in the presentation during the experiment), (log) frequency (from LEXESP corpus^37^), and (inverse of) word-length were used based on previous analyses^20^. The positions within the sentence, the line, and the text were rescaled to the [0 1] interval and named as a relative position in the sentence (rps), relative position in the line (rpl), and relative position in the text (rpt). All covariates were centered so that the intercept estimated the mean log duration. The baseline model fitted is presented in Eq. (3). The * means that in addition to the main effect of length and frequency the interaction between them was tested. The last three effects with the syntax (1∣*XXXX*) are the random effects.

3 $$\begin{array}{rcl}log(FPRT) &  \sim  & LaunchSite+Length* Frequency+rpl+rpt\\  &  & +\ rps+(1| sujid)+(1| textid)+(1| wordid)\end{array}$$

For each fixed effect t-values (estimated slope over standard deviation) were being reported. There is no clear definition of "degree of freedom” for LMMs^21^, and therefore precise p values cannot be estimated. However, in general, given the large number of observations, subjects, sentences, and words considered in our analysis and the comparatively small number of fixed and random effects estimated, the t-distribution is equivalent to a normal distribution for all practical purposes (i.e., the contribution of the degrees of freedom to the test statistic was negligible). Our criterion for referring to an effect as significant was *t* = *b*/*SE* > 2.0.

To further analyze how the computer estimations of the predictability were used in the fitted LMMs, remotion of predicted effects using the function *remef()*^24^ was performed. This function subtracted from the dependent variable the predicted values using the indicated variables. In the present study all the fixed effects were removed and the residuals were used to fit a new model with the same random factors and the cloze-Predictability. Analyzing the significance of the cloze-Predictability in this new model and comparing it with the significance of the **M1.N** (baseline model + cloze-Predictability) produced an idea of how similar the computational-Predictability was in relation to cloze-Predictability.

Quantile-quantile plots (Supplementary Fig. S3A,B) and residuals plots (Supplementary Fig. S3C,D) did not show significant deviance from the model assumptions.

## Supplementary information

Supplementary Information.

## Data Availability

The datasets analyzed for this study can be found at http://reading.liaa.dc.uba.ar. More detailed or complementary data are available on request.
