# Supplementary material for: Human and computer estimations of Predictability of words in written language
Source: Sci Rep. 2020 Mar 10;10:4396. doi: 10.1038/s41598-020-61353-z (PMC7064512; doi:10.1038/s41598-020-61353-z)
Supplement: Supplementary file 1 — Supplementary Information. [file 41598_2020_61353_MOESM1_ESM.pdf]

Human and computer estimations of Predictability of words in written language

Bruno Bianchi[1,*], **Gastón Bengolea Monzón**[1], **Luciana Ferrer**[1], **Diego Fernández Slezak**[1,2], **Diego E. Shalom**[3], **and Juan E. Kamienkowski**[1,3]

[1]Laboratorio de Inteligencia Artificial Aplicada, Instituto de Ciencias de la Computación, Facultad de Ciencias Exactas y Naturales, Universidad de Buenos Aires - Consejo Nacional de Investigación en Ciencia y Técnica (Argentina)
[2]Departamento de Computación, Facultad de Ciencias Exactas y Naturales, Universidad de Buenos Aires (Argentina)
[3]Departamento de Física, Facultad de Ciencias Exactas y Naturales, Universidad de Buenos Aires (Argentina)
[*]bbianchi@dc.uba.ar

SUPPLEMENTARY INFORMATION

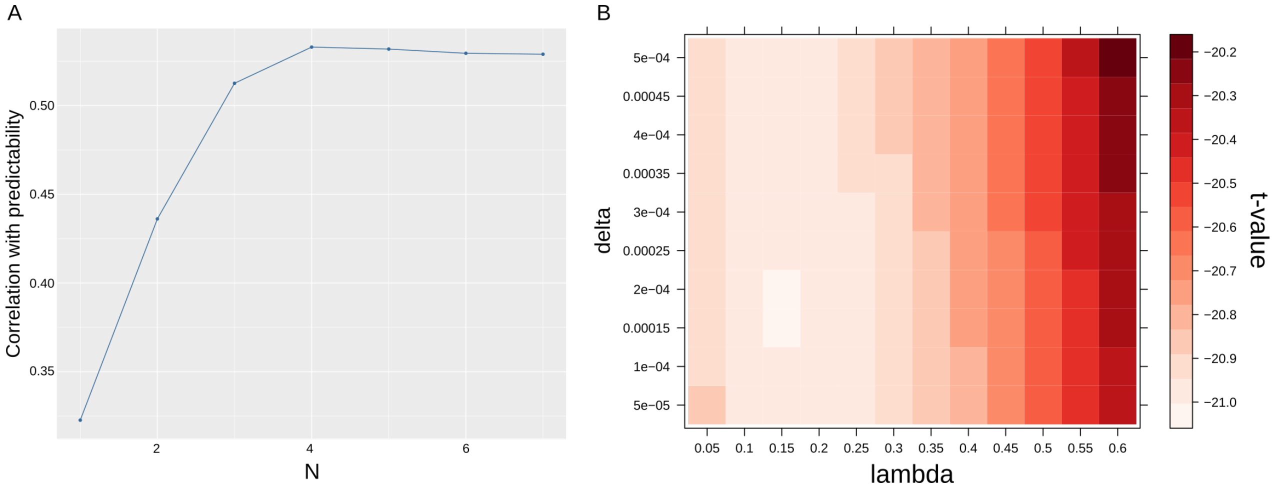

Supplementary Figure S1. N-gram parameter exploration. **A)** Correlation of the N-gram probability with the (*logit*) cloze-Predictability as a function of N. **B)** t-value of the 4-gram+cache probability in the baseline Linear Mixed Model as a function of the mixing parameter between the 4-gram and the cache (λ) and the smoothing parameter of cache (δ).

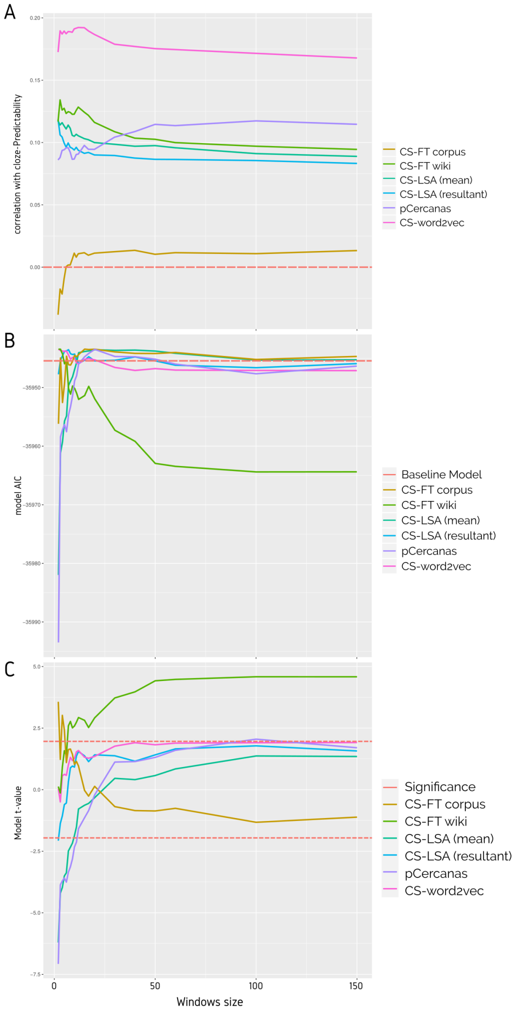

Supplementary Figure S2. Parameter exploration of word embeddings (context size (number of words)) using different measures. **A)** Correlation between the similarity of the context and the (*logit*) cloze-Predictability. **B)** AIC of a LMM similar to M3.N or M4.N. **C)** t-value of the explored Word embedding co-variable in the same LMM.

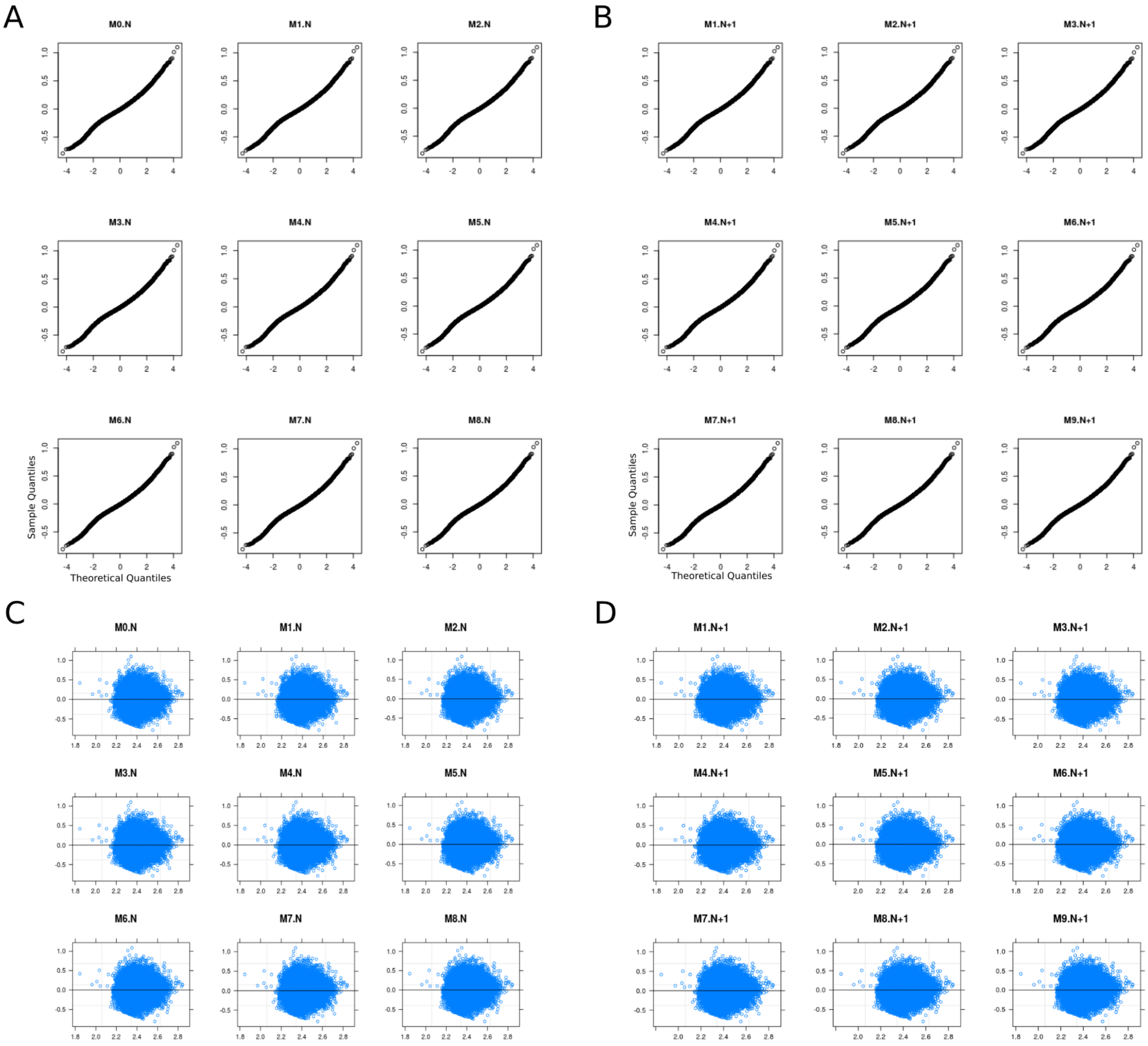

Supplementary Figure S3. Analyses of model assumptions (A) Quantile - Quantile plots and (C) Residual plots for the fitted models of the Gaze duration (word N), including the cloze-Predictability of the word N, and different combinations of the NLP algorithms for the N. (B) Quantile - Quantile plots and (D) Residual plots for the fitted models of the Gaze duration (word N), including the cloze-Predictability of the word N and N+1, and different combinations of the NLP algorithms for the N and N+1 (next). Note: M0.N+1 is not shown because ir is the same model as M0.N.

	M0.N		M1.N		M2.N		M3.N		M4.N		M5.N		M6.N		M7.N		M8.N	
	Estimate	CI	Estimate	CI	Estimate	CI	Estimate	CI	Estimate	CI	Estimate	CI	Estimate	CI	Estimate	CI	Estimate	CI
(Intercept)	2.3694	[2.3526 2.3862]	2.3688	[2.3527 2.385]	2.3171	[2.3 2.3341]	2.3748	[2.3573 2.3923]	2.3450	[2.3251 2.3649]	2.3152	[2.2973 2.333]	2.2881	[2.2682 2.3081]	2.3493	[2.3292 2.3693]	2.2888	[2.2687 2.309]
freq	-0.0207	[-0.0244 -0.0169]	-0.0194	[-0.023 -0.0158]	-0.0038	[-0.0077 0.0001]	-0.0183	[-0.0226 -0.0141]	-0.0222	[-0.0259 -0.0184]	-0.0044	[-0.0087 -0.0002]	-0.0054	[-0.0093 -0.0015]	-0.0190	[-0.0232 -0.0147]	-0.0050	[-0.0093 -0.0008]
invlength	-0.4905	[-0.5435 -0.4375]	-0.4801	[-0.5308 -0.4293]	-0.4855	[-0.5353 -0.4356]	-0.4855	[-0.5387 -0.4322]	-0.4734	[-0.5267 -0.4201]	-0.4870	[-0.537 -0.4369]	-0.4655	[-0.5156 -0.4155]	-0.4634	[-0.517 -0.4097]	-0.4643	[-0.5147 -0.4139]
invlength:freq	0.4087	[0.3615 0.4559]	0.3951	[0.35 0.4402]	0.3562	[0.3117 0.4008]	0.4130	[0.3656 0.4604]	0.3941	[0.3467 0.4416]	0.3546	[0.3099 0.3994]	0.3387	[0.294 0.3834]	0.3982	[0.3508 0.4458]	0.3394	[0.2946 0.3842]
Nlaunchsite	0.0352	[0.0336 0.0367]	0.0352	[0.0337 0.0368]	0.0346	[0.0331 0.0362]	0.0351	[0.0336 0.0367]	0.0352	[0.0336 0.0367]	0.0346	[0.0331 0.0362]	0.0346	[0.0331 0.0362]	0.0351	[0.0336 0.0367]	0.0346	[0.0331 0.0362]
rpl	0.0181	[0.0095 0.0267]	0.0172	[0.0087 0.0257]	0.0187	[0.0102 0.0272]	0.0185	[0.0099 0.0271]	0.0180	[0.0095 0.0266]	0.0186	[0.0101 0.027]	0.0186	[0.0102 0.0271]	0.0186	[0.01 0.0271]	0.0187	[0.0102 0.0271]
rps	-0.0169	[-0.0231 -0.0107]	-0.0151	[-0.0212 -0.009]	-0.0179	[-0.024 -0.0118]	-0.0167	[-0.0229 -0.0105]	-0.0167	[-0.0228 -0.0105]	-0.0180	[-0.024 -0.0119]	-0.0176	[-0.0237 -0.0116]	-0.0164	[-0.0225 -0.0102]	-0.0176	[-0.0237 -0.0115]
rpt	-0.0118	[-0.0177 -0.0059]	-0.0098	[-0.0156 -0.0039]	-0.0115	[-0.0173 -0.0057]	-0.0119	[-0.0178 -0.006]	-0.0120	[-0.0179 -0.0061]	-0.0114	[-0.0172 -0.0056]	-0.0117	[-0.0174 -0.0059]	-0.0121	[-0.018 -0.0062]	-0.0117	[-0.0175 -0.0059]
cloze-Pred	–	–	-0.0301	[-0.0337 -0.0264]	–	–	–	–	–	–	–	–	–	–	–	–	–	–
4-gram+cache	–	–	–	–	-0.0175	[-0.0191 -0.0158]	–	–	–	–	-0.0175	[-0.0192 -0.0159]	-0.0176	[-0.0193 -0.016]	–	–	-0.0176	[-0.0192 -0.0159]
CS-LSA (w=9)	–	–	–	–	–	–	-0.0316	[-0.0602 -0.0031]	–	–	0.0098	[-0.0184 0.038]	–	–	-0.0468	[-0.0759 -0.0176]	-0.0060	[-0.0348 0.0227]
CS-FT (w=50)	–	–	–	–	–	–	–	–	0.0586	[0.0326 0.0846]	–	–	0.0681	[0.0434 0.0929]	0.0674	[0.0409 0.094]	0.0692	[0.0439 0.0945]

Supplementary Table S3. Estimates and Confidence Intervals (CI) (2.5% and 97.5%) for LMM analysis of the Gaze duration (word N), including the cloze-Predictability of the word N, and different combinations of the NLP algorithms for the N: 4-gram+cache, LSA (with a context of nine words)and FastText (with a context of 50 words) (See Figure 3).

	M0.N+1		M1.N+1		M2.N+1		M3.N+1		M4.N+1		M5.N+1		M6.N+1		M7.N+1		M8.N+1		M9.N+1	
	Estimate	CI	Estimate	CI	Estimate	CI	Estimate	CI	Estimate	CI	Estimate	CI	Estimate	CI	Estimate	CI	Estimate	CI	Estimate	CI
(Intercept)	2.3694	[2.3526 2.3862]	2.3689	[2.3528 2.385]	2.3175	[2.3003 2.3347]	2.3797	[2.362 2.3974]	2.3578	[2.3369 2.3788]	2.3301	[2.3111 2.3491]	2.3058	[2.2841 2.3276]	2.3613	[2.3402 2.3824]	2.3122	[2.2899 2.3345]	2.3288	[2.3104 2.3471]
freq	-0.0207	[-0.0244 -0.0169]	-0.0194	[-0.023 -0.0158]	-0.0038	[-0.0077 0.0001]	-0.0189	[-0.0232 -0.0146]	-0.0224	[-0.0262 -0.0187]	-0.0056	[-0.0099 -0.0013]	-0.0058	[-0.0097 -0.0019]	-0.0195	[-0.0238 -0.0153]	-0.0062	[-0.0104 -0.0019]	-0.0041	[-0.0079 -0.0002]
invlength	-0.4905	[-0.5435 -0.4375]	-0.4797	[-0.5304 -0.429]	-0.4854	[-0.5352 -0.4355]	-0.4872	[-0.5402 -0.4341]	-0.4747	[-0.5279 -0.4214]	-0.4888	[-0.5386 -0.439]	-0.4667	[-0.5166 -0.4166]	-0.4656	[-0.5191 -0.412]	-0.4671	[-0.5173 -0.4168]	-0.4869	[-0.5367 -0.4371]
invlength:freq	0.4087	[0.3615 0.4559]	0.3945	[0.3494 0.4396]	0.3563	[0.3117 0.4008]	0.4100	[0.3627 0.4573]	0.3909	[0.3436 0.4383]	0.3506	[0.3061 0.3952]	0.3358	[0.2911 0.3804]	0.3944	[0.347 0.4418]	0.3348	[0.2901 0.3795]	0.3539	[0.3094 0.3985]
Nlaunchsite	0.0352	[0.0336 0.0367]	0.0352	[0.0336 0.0367]	0.0346	[0.0331 0.0362]	0.0351	[0.0336 0.0367]	0.0352	[0.0336 0.0367]	0.0346	[0.0331 0.0362]	0.0346	[0.0331 0.0362]	0.0351	[0.0336 0.0367]	0.0347	[0.0331 0.0362]	0.0346	[0.0331 0.0362]
rpl	0.0181	[0.0095 0.0267]	0.0169	[0.0084 0.0254]	0.0188	[0.0103 0.0273]	0.0179	[0.0093 0.0265]	0.0174	[0.0088 0.026]	0.0185	[0.0101 0.027]	0.0184	[0.0099 0.0268]	0.0178	[0.0092 0.0264]	0.0184	[0.01 0.0269]	0.0181	[0.0097 0.0266]
rps	-0.0169	[-0.0231 -0.0107]	-0.0147	[-0.0209 -0.0086]	-0.0179	[-0.024 -0.0118]	-0.0163	[-0.0224 -0.0101]	-0.0165	[-0.0226 -0.0103]	-0.0173	[-0.0234 -0.0112]	-0.0174	[-0.0235 -0.0114]	-0.0160	[-0.0222 -0.0098]	-0.0170	[-0.023 -0.0109]	-0.0177	[-0.0238 -0.0116]
rpt	-0.0118	[-0.0177 -0.0059]	-0.0095	[-0.0153 -0.0037]	-0.0115	[-0.0173 -0.0057]	-0.0122	[-0.0181 -0.0063]	-0.0121	[-0.018 -0.0062]	-0.0119	[-0.0177 -0.0061]	-0.0118	[-0.0176 -0.006]	-0.0123	[-0.0182 -0.0064]	-0.0121	[-0.0179 -0.0063]	-0.0116	[-0.0174 -0.0057]
cloze-Pred	–	–	-0.0300	[-0.0336 -0.0263]	–	–	–	–	–	–	–	–	–	–	–	–	–	–	–	–
cloze-Pred next	–	–	-0.0032	[-0.0061 -0.0003]	–	–	–	–	–	–	–	–	–	–	–	–	–	–	–	–
4-gram+cache	–	–	–	–	-0.0175	[-0.0191 -0.0159]	–	–	–	–	-0.0175	[-0.0192 -0.0159]	-0.0176	[-0.0193 -0.016]	–	–	-0.0176	[-0.0192 -0.0159]	-0.0174	[-0.019 -0.0158]
4-gram+cache next	–	–	–	–	0.0002	[-0.0008 0.0013]	–	–	–	–	0.0026	[0.0011 0.0041]	0.0012	[0.0001 0.0024]	–	–	0.0026	[0.0012 0.0041]	–	–
CS-LSA (w=9)	–	–	–	–	–	–	-0.0250	[-0.0538 0.0038]	–	–	0.0205	[-0.008 0.049]	–	–	-0.0424	[-0.0718 -0.0129]	0.0025	[-0.0266 0.0316]	–	–
CS-LSA next (w=9)	–	–	–	–	–	–	-0.0206	[-0.0324 -0.0089]	–	–	-0.0377	[-0.0539 -0.0215]	–	–	-0.0111	[-0.0252 0.0029]	-0.0276	[-0.0451 -0.0101]	–	–
CS-FT (w=50)	–	–	–	–	–	–	–	–	0.0600	[0.034 0.0859]	–	–	0.0692	[0.0445 0.094]	0.0677	[0.0411 0.0942]	0.0681	[0.0429 0.0934]	–	–
CS-FT next (w=50)	–	–	–	–	–	–	–	–	-0.0261	[-0.0396 -0.0125]	–	–	-0.0301	[-0.0449 -0.0152]	-0.0186	[-0.0349 -0.0024]	-0.0204	[-0.0364 -0.0043]	-0.0224	[-0.0358 -0.009]

Supplementary Table S3. Estimates and Confidence Intervals (CI) (2.5% and 97.5%) for LMM analysis of the Gaze duration (word N), including the cloze-Predictability of the word N and N+1, and different combinations of the NLP algorithms for the N and N+1 (next): 4-gram+cache, LSA (with a context of nine words)and FastText (with a context of 50 words) (See Figure 4).
